# fNIRS improves seizure detection in multimodal EEG-fNIRS recordings

**DOI:** 10.1117/1.JBO.24.5.051408

**Published:** 2019-02-07

**Authors:** Parikshat Sirpal, Ali Kassab, Philippe Pouliot, Dang Khoa Nguyen, Frédéric Lesage

**Affiliations:** a Université de Montréal, École Polytechnique de Montréal, Montréal, Québec, Canada; b Neurology Division, Centre Hospitalier de l’Université de Montréal, Montréal, Québec, Canada; c Montreal Heart Institute, Research Centre, Montreal, Québec, Canada

**Keywords:** electroencephalography-functional near-infrared spectroscopy, functional brain imaging, deep neural networks, epilepsy, seizure detection

## Abstract

In the context of epilepsy monitoring, electroencephalography (EEG) remains the modality of choice. Functional near-infrared spectroscopy (fNIRS) is a relatively innovative modality that cannot only characterize hemodynamic profiles of seizures but also allow for long-term recordings. We employ deep learning methods to investigate the benefits of integrating fNIRS measures for seizure detection. We designed a deep recurrent neural network with long short-term memory units and subsequently validated it using the CHBMIT scalp EEG database—a compendium of 896 h of surface EEG seizure recordings. After validating our network using EEG, fNIRS, and multimodal data comprising a corpus of 89 seizures from 40 refractory epileptic patients was used as model input to evaluate the integration of fNIRS measures. Following heuristic hyperparameter optimization, multimodal EEG-fNIRS data provide superior performance metrics (sensitivity and specificity of 89.7% and 95.5%, respectively) in a seizure detection task, with low generalization errors and loss. False detection rates are generally low, with 11.8% and 5.6% for EEG and multimodal data, respectively. Employing multimodal neuroimaging, particularly EEG-fNIRS, in epileptic patients, can enhance seizure detection performance. Furthermore, the neural network model proposed and characterized herein offers a promising framework for future multimodal investigations in seizure detection and prediction.

## 1 Introduction

Continuous video-electroencephalography (EEG) surveillance is often used in hospitals to monitor patients at high-risk of epileptic seizures,^1^ particularly patients with drug-resistant chronic epilepsy admitted in epilepsy-monitoring units or critically ill patients admitted to the intensive care unit after an acute brain injury, such as stroke, head trauma, brain hemorrhage, or brain infection. While some seizures are clinically evident, such as generalized tonic–clonic seizures, others are subtle in terms of clinical manifestations (e.g., subtle facial or limb twitches), for which recognition by EEG is particularly well suited. Moreover, some seizure events are purely electrical being only detectable on EEG or even completely asymptomatic (from isolated electrical seizures to nonconvulsive status). Nonconvulsive status epilepticus (defined as a continuous state of seizures without convulsions or multiple nonconvulsive seizures for more than 30 min without interictal full recovery) has been found to account for up to 20% of all cases of status epilepticus in general hospitals and up to 47% in the intensive care unit.^2^ Functional near-infrared spectroscopy (fNIRS) has emerged as a safe and noninvasive optical technique that exploits neurovascular coupling to indirectly measure brain activity. Measured relative changes in both oxygenated and deoxygenated hemoglobin can be used to assess cortical activation during overt and subtle seizures.^3^ Continual fNIRS cerebral monitoring provides the ability to track regional oxygenation changes before, during, and after these ictal events.^4^^–^^7^ In recent years, multimodal approaches have emerged integrating EEG with fNIRS to offer dual hemodynamic and electro-potential characterization of a seizure event,^8^^–^^10^ whereas EEG data record the macroscopic temporal change in brain electrical activity; fNIRS approximates brain hemodynamic changes via spectroscopic measurements of oxyhemoglobin (HbO) and deoxyhemoglobin (HbR). fNIRS depends on the slow dynamics of the hemodynamic response, thereby yielding lower temporal resolution. According to literature and with the optode spacing used in this work, NIRS yields a spatial resolution of ∼1  cm.^11^ Given the different characteristics and physiological information provided by each modality, multimodal EEG-fNIRS data provide complementary electrical and hemodynamic information, which may be exploited to implement appropriate diagnostic and treatment strategies.

Seizure detection has traditionally been approached using EEG with quantitative feature signal processing techniques, such as the application of Fourier transform analysis (FFT), wavelet transforms, and spectral decompositions.^12^^,^^13^ Briefly, EEG FFT analysis allows for the convenient processing of lengthy and noisy recordings in the frequency domain, allowing hidden features within the time series to become apparent. Wavelet analysis can be thought as an extension of the Fourier transform that works on a multiscale basis instead of on a single scale either in time or frequency. This multiscale feature of the wavelet transform allows coarse to fine time-frequency signal resolution analysis of the signal. Using the above features as input, artificial neural networks (ANN) have been used and emerged as better models than traditional techniques for seizure detection if appropriate processing of data occurs a-priori.^14^ Traditionally, researchers have used ANNs as a final step to classify hand-engineered features.^15^^–^^17^ In contrast to the process of hand-engineering features, deep learning (DL) methodologies learn intrinsic data features to obtain relevant data abstractions.^18^ DL models have been used for seizure detection using EEG data streams,^19^^–^^22^ in which impressive metric scores were achieved. This work aims to investigate for the first time the additional benefit that hemodynamic information derived from fNIRS recordings provides in a seizure detection task in the context of multimodal EEG-fNIRS recordings. Our work exploits artificial intelligence models, particularly the long short-term memory (LSTM) unit, on human epilepsy data without performing extensive feature extraction, selection, or signal preprocessing.

## 2 Methods

### 2.1 Patient Recruitment, Characterization, and Seizure Types

Forty patients between the ages of 11 and 62 years with refractory focal epilepsy admitted to the epilepsy-monitoring unit to record their seizures (and determine if they could be good candidates for epilepsy surgery) were recruited for this study. The ethics committees of Sainte-Justine and CHUM Notre-Dame Hospitals approved the study, and informed consent was obtained from all subjects. Patient inclusion criteria primarily consisted of the following: patient (or parental) consent and focal epilepsy confirmed by clinical history, electroencephalographic, and/or imaging findings. Exclusion criteria included the following: subjects with significant progressive disorders or unstable medical conditions. Patients underwent a full physical exam, an anatomical magnetic resonance brain image (MRI), positron emission tomography, ictal single-photon computed tomography, and a magnetoencephalography study. Subsequently, continuous EEG-fNIRS recordings were performed at the Optical Imaging Laboratory of Sainte-Justine Hospital, Montreal, Canada. An epileptologist was available at all times to ensure patient safety and inspected data for congruency with clinical semiology analysis, the location of scalp EEG findings, and location of the epileptogenic lesion on MRI if present. The data corpus collected included 266 epileptiform abnormalities in total. Of these, 89 were seizures, the majority of which were temporal lobe seizures followed by frontal lobe seizures. The remainder of the dataset included interictal epileptiform discharges and periodic epileptiform discharges. Seizure duration ranged from 5.1 to 62 s with an average of 21.7 s. The details concerning patients, seizure types, MRI findings, and foci seen using EEG and fNIRS modalities used in this study are detailed in Table 1.

**Table 1:** Clinical profiles of refractory epilepsy patients.

Patient	Age, sex	Total recordings	Epilepsy classification	MRI findings	EEG focus	fNIRS focus
1	11, M	9	R FLE	N	RF	RF
2	21, M	11	L FLE	N	LF	Bi-F (L > R)
3	13, F	2	R FPLE	N	LP	LP
4	35, F	4	R FLE	N	RF	RF
5	25, F	5	R FLE	N	LF	LF
6	16, M	7	L FLE	RF encephalomalacia	LF	PF
7	63, M	5	L TLE	N	LT	LT
8	47, F	3	R LNTLE	N	Bi-T	LT
9	23, M	5	R FLE	N	RF	RF
10	43, M	8	R FLE	RF encephalomalacia	RF	RF
11	19, F	4	L MBTLE	N	RT	RT
12	45, M	7	R FLE	N	Bi-F (R > L)	Bi-F
13	38, F	1	L LNTLE	N	LF	LFT
14	53, F	11	L LFPLE	N	LFP	Bi-F (L > R)
15	24, M	6	L LNTLE	N	RT	RT
16	31, M	3	Bi-MBTLE	R HA	Bi-T (R > L)	RT
17	31, M	11	R LNTLE	N	RT	RT
18	23, M	6	R FPLE	RF CD	RF	RF
19	27, M	3	R FLE	N	RF	RF
20	21, M	11	R FLE	RHA	RT	RF
21	50, M	6	L MBTLE	LHA	Bi-F	RF
22	38, F	5	R LNTLE	N	RT	RT
23	34, M	10	L LNTLE	N	LT	LT
24	56, M	7	R FLE	N	RF	RF
25	11, M	4	R LNTLE	N	RT	RT
26	43, M	5	L LPTLE	N	LT	LP
27	24, M	3	R FLE	N	RF	RF
28	46, M	7	L FLE	N	LF	LF
29	30, F	5	L LNTLE	N	LT	LT
30	62, F	6	L FLE	N	LF	LF
31	43, M	8	L FLE	N	LF	LF
32	13, M	6	Bi-LNTLE	N	Bi-T	Bi-T
33	22, M	5	R FLE	N	RF	RF
34	25, M	7	R FLE	N	RF	RF
35	28, M	9	L FLE	N	LF	LF
36	44, F	7	R FLE	N	RF	RF
37	49, M	3	R FLE	N	RF	RF
38	32, M	2	R FLE	N	RF	RF
39	19, F	4	R FLE	N	RF	RF
40	19, F	3	R FLE	N	RF	Bi-F (R > L)

Note: F, female; M, male; FLE, frontal lobe epilepsy; FPLE, fronto-parietal lobe epilepsy; OLE, occipital lobe epilepsy; NTLE, neocortical temporal lobe epilepsy; MTLE, mesial temporal lobe epilepsy; RF, right frontal, LF, left frontal, Bi, bilateral, P, parietal. F, frontal, P, parietal, N, normal, L, left, R, right; HA, hippocampal atrophy, CD, cortical dysplasia, RHA, right hippocampal atrophy, and LHA, left hippocampal atrophy.

### 2.2 EEG-fNIRS Instrumentation and Data Acquisition

The EEG-fNIRS instrumentation included the use of custom helmets designed to mount a total of 80 optical fibers (64 light sources in pairs for both wavelengths and 16 detectors) and 19 carbon EEG electrodes. First, the EEG data recording system was installed according to the traditional 10–20 system. Following this, we installed custom-made helmet-holding optical fibers. The installation time, including hair removal, patient positioning, adjustment of signal intensity, and optode repositioning, typically was between 1 and 2 h. A description of our setup and its near full-head coverage is provided in previous publications.^8^^,^^9^ Optode and electrode positions were coregistered onto three-dimensional (3-D) high-resolution anatomical MRI images using neuro-navigation (Brainsight, Rogue-Research Inc.). The EEG data stream was recorded at 500 Hz with a Neuroscan Synamps 2TM system (Compumedics). To remove instrumental noise, bandpass filtering between 0.1 and 100 Hz was applied. Simultaneously, the fNIRS data stream was acquired using a multichannel frequency-domain system at 19.5 Hz (Imagent Tissue Oximeter, ISS Inc., Champaign, Illinois) with wavelengths of 690 and 830 nm for sensitivity to HbR and HbO, respectively. The channel positions were cross-referenced with the MRI and were adapted to ensure coverage of the epileptic focus, the contralateral homologous region, and as much area as possible of the other lobes. Data were acquired for 2 to 12 consecutive sessions of 15 min while the patient was in a resting state. Multiple sessions of data acquisition were performed since during a single acquisition; seizure events are not sure to occur. Sensitivity of near-infrared light to cortical tissue was maintained by positioning the optical channels ∼3 to 4 cm apart. During installation, we verified channel quality using signal intensity.

### 2.3 Seizure Identification

The EEG tracing was analyzed using Analyzer 2.0 (Brain Products GmbH, Germany) by a certified clinical neurophysiologist and reviewed by an epileptologist to identify interictal epileptiform discharges and seizures. Seizures were marked in the presence of a transient electrographic rhythmic discharge evolving in amplitude, frequency, and spatial distribution changes associated with stereotypical seizure semiology on video.

### 2.4 Data Processing and Analysis

As mentioned in Sec. 2.1, recordings obtained from known epileptic patients were evaluated for seizure occurrence, leading to a compendium of 200 recordings totaling 50 h of recording time and 89 seizure events lasting in duration from a few seconds to ∼1  min. An average time offset of 4.5 s was used between modalities to feed the neural network corresponding to the average time delay between neural activity and the hemodynamic response.^23^ Prior to analysis, each channel was further verified for signal quality (intensity and presence of physiology, e.g., heart beat). Channels that did not have good signal were eliminated from the analysis. For each recording, distinct seizure and nonseizure classes were partitioned from the data. Entire seizure segments were extracted and nonseizure segments were subsequently defined as those data points that do not overlap with seizure segments. Postacquisition, raw data were processed using the HomER package^24^ (Photon Migration Imaging Lab; Massachusetts General Hospital, Boston, Massachusetts) to convert raw fNIRS data into hemodynamic parameters, namely oxygenated and deoxygenated hemoglobin.^25^ In our analyses, the modified Beer–Lambert law was used to relate light attenuation to changes in absorption and enable the estimation of changes in oxygenated and deoxygenated hemoglobin as they vary in space and time.

### 2.5 Deep Neural Networks

To a large extent, human seizure activity is highly unpredictable. Longitudinal analyses suggest temporal and spatial irregularities to be intrinsic to seizure activity. Recurrent neural networks (RNNs) have become state of the art for sequence modeling and generation.^26^ The “LSTM unit” is a popular variant of RNNs with proven ability to generate sequences in various applications, particularly text and sequence processing.^27^^,^^28^ LSTM models learn important past behaviors due to their innate ability to learn from and remember previous time steps and their important features. LSTM units hold an advantage over other methods in modeling long-term dependencies due to automatically learned “input,” “output,” and “forget” gates. The success of RNNs in these domains motivated our work of applying LSTM-RNNs for human seizure activity detection in multimodal EEG-fNIRS recordings.

### 2.6 Model Architecture

**Fig. 1:**
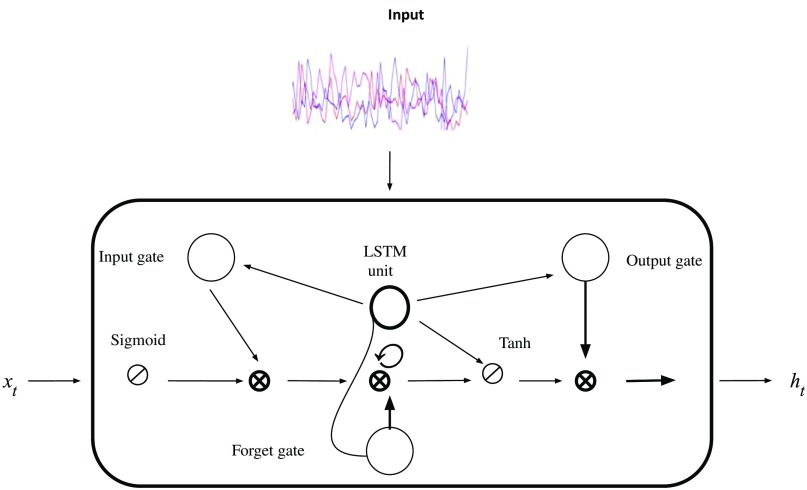
LSTM unit structure. The input is fed into LSTM units with 64 hidden units followed by a final dense layer. The input gate decides which values will be updated and creates a vector of new values to be added and updated to the state. After data input, the LSTM’s forget gate decides which information to discard. This gate examines the prior hidden state (h) and current input, yielding a binary output. Subsequently, the LSTM decides what new information to store in the cell state. Finally, the LSTM unit decides sequential output, which is based on the current cell state. The sigmoid and hyperbolic activation functions determine which parts of the cell state to output.

In this section, we describe the vanilla LSTM model architecture that was used to perform the seizure detection task. The architecture consists of: (1) input layer, (2) LSTM units, and (3) a dense layer. Figure 1 shows the LSTM unit structure, featuring input, forget, and output gates. We designed our architecture to use the following activation functions: (1) hyperbolic tangent for the LSTM units and (2) logistic sigmoid for the gates. Softmax, categorical cross-entropy was used as the loss function, with alpha = 0.95, since it is well suited for categorization problems.^18^ The hyperparameters, shown in Table 2, used to train our model were heuristically tuned to achieve sufficient performance and we validated our results by comparing performance with other techniques developed in the literature (Table 3). The hidden state, $h_{t}$, is an element-wise application of the sigmoid function.^27^^,^^28^ The output of each block is recurrently connected back to the input and the gates. Our model generates subsequent data sequences according to the following two steps:

1. At every time step, the LSTM layer receives input, $x_{t}$. Inputs to the LSTM cell include the previous hidden state and the previous memory state.
2. The LSTM layers then produce output, which is used to sample a new set of input variables $x_{t+1}$. The outputs from the LSTM cell are the current hidden state and the current memory state.

**Table 2:** LSTM-RNN heuristic hyperparameters.

Hyperparameters	Value	Method
Learning rate	$1\times 10^{-3}$	Adam
Epochs	100	Experimental
Batch size	784	Experimental
LSTM units	10	Experimental

**Table 3:** A comparison of selected studies in the automated detection of seizure using EEG signals from the Bonn and CHBMIT databases.

Author	Year	Database	Research innovation	Neural network architecture	Performance (%)
Ghosh-Dastidar et al.^29^	2007	Bonn	Wavelet-chaos	ANN	Accuracy = 96.7
Shoeb et al.^15^	2004	CHBMIT	SVM	ANN	Accuracy = 96
Chua et al.^30^	2009	Bonn	Entropy feature determination	Gaussian mixture models	Accuracy = 93.1 Sensitivity = 89.7 Specificity = 94.8
Acharya et al.^31^	2017	Bonn	Ten-fold cross validation	CNN	Accuracy = 88.7 Sensitivity = 95.0 Specificity = 90
Shoeb et al.^16^	2009	CHBMIT	Patient-specific detection	ANN, SVM	Accuracy = 96
Martis et al.^32^	2012	Bonn	Empirical mode decomposition (Hilbert–Huang transformation)	Decision trees	Accuracy = 95.3 Sensitivity = 98.0 Specificity = 97.0
Guo et al.^33^	2011	Bonn	Genetic programming	ANN with k-nearest neighbors	Accuracy = 93.5
Bhattacharyaa et al.^34^	2017	Bonn	Tunable Q-factor wavelet transform	ANN, SVM	Accuracy = 99.4 Sensitivity = 97.9 Specificity = 99.5
This work	2018	CHBMIT	Validation of LSTM-RNN model	LSTM-RNN	Accuracy = 98.2 Sensitivity = 95.9 Specificity = 92.1

Note: AAN, artificial neural network; CNN, convolutional neural network; SVM, support vector machine; and LSTM-RNN, long short-term memory RNNs.

Input data are transformed into a 3-D tensor with standard dimensions of an LSTM-RNN.^18^ The final gradients are back propagated at each time step with adaptive moment estimation as an optimizer for stochastic gradient descent. The prediction is a binary output derived from the softmax function.^18^

Adaptive moment estimation and dropout on nonrecurrent connections^35^^–^^37^ were utilized to regularize our model to avoid overfitting.

### 2.7 Network Training and Model Validation

A feed dictionary was generated, and for each step, mini-batches of training examples were presented to the network. Input data were binary partitioned into appropriate seizures and nonseizures segments. Using these newly segmented classes as input, our network was trained to compute scores for seizure and nonseizure segments. Truncated back propagation through time, a modified form of the conventional back propagation through time (BPTT) training algorithm for RNNs,^38^ was used for training. Briefly, BPTT works to unroll the RNN and backward propagate the error between the expected output and the obtained output for a given input. The weights are then updated with the accumulated gradients. We first validated our model using k-fold cross validation (k=10), on the standard CHBMIT scalp EEG dataset^16^^,^^39^ as this dataset is vast, and the seizures contained within are of long duration. Following this, we aimed to test our hypothesis regarding the relevance of adding fNIRS data for improving the task of seizure detection using our database. Stand-alone in-house EEG data, followed by stand-alone fNIRS data, and finally multimodal data were used as input for the network. Our models were implemented on two NVIDIA TITAN X GPUs with 12 GB memory using the Keras platform with Tensorflow backend for a total training time of 10 h.

## 3 Experimental Results

This section describes the model validation results using the standard CHBMIT database, and finally, statistical analyses of the model using our in-house datasets are described.

### 3.1 Model Validation on the CHBMIT Database

The CHBMIT dataset includes 198 seizures from 22 patients. To evaluate performance, we defined the following metrics:

$$\text{Accuracy}=\frac{\text{True positives }+\text{ True negatives}}{\text{Total number of examples}}.$$ (1)

Sensitivity, also known as recall, measures the proportion of actual positives that are correctly identified. Specificity, also called the true negative rate, measures the model’s performance at classifying negative observations. False positive rate is defined as

$$\text{False positive rate}=\frac{\text{False positives}}{\text{Total number of negative examples}}.$$ (2)

Precision is also known as the positive predictive value and is defined as

$$\text{Precision}=\frac{\text{True positives}}{\text{Total number of positive examples}}.$$ (3)

Using this dataset, our model derived performance metrics of accuracy, sensitivity, specificity, and false positive rate of 98.2%, 95.9%, 92.1%, and 2.9%, respectively. The validation results of our network using the CHBMIT corpus are shown in Tables 3 and 4. Table 4 details our model’s performance on both the CHBMIT standard dataset and our in-house EEG data. It should be noted that accuracy is the most intuitive performance measure and works best when there is symmetry in the available data. In our experiments, we assumed the seizure state to be a rare state^40^ and used more representative parameters to evaluate performance.

**Table 4:** Performance results for EEG data derived from the CHBMIT dataset and our in-house EEG data.

Data	Epochs	Mean accuracy (%)	ROC
CHBMIT EEG	100	98.20	0.94
In-house EEG	100	97.60	0.90

The algorithms were further validated with in-house data. Focusing first on stand-alone EEG data we observed no significant difference in learning rate when comparing to the CHBMIT dataset. Studying the integration of measurement type, performance scores steadily increased for each data type: EEG, fNIRS, and EEG-fNIRS. The performance of the proposed model with respect to each data type is summarized in Table 4. Monitoring cross-entropy loss ensured network generalization.

### 3.2 Model Evaluation

This section presents the classification results for seizure and nonseizure classes using our in-house datasets. Our evaluation uses all of the seizure and nonseizure blocks from all subjects and all recordings. To estimate performance of our model on unseen data, we utilized statistical methods derived from k-fold cross validation. The dataset was randomly shuffled and subsequently split it into “k” groups. The model was fit on the training set and evaluated on the test set, yielding an evaluation score. Each fold has data points from a subset of patients, chosen randomly from “k=10” folds. The data were preshuffled to allow for randomization and we sequentially instantiated k identical models and trained each one on “k−1” partitions while evaluating on the remaining data.

Performance metrics on all measures improved in EEG-fNIRS data as compared to either EEG or fNIRS alone. From our cross-validation results, we notice that multimodal data consistently perform better as compared to either stand-alone EEG or fNIRS data. Mean squared error between EEG and fNIRS recordings was determined to be 0.61 and between EEG and multimodal recordings to be 0.79. Likewise, the mean absolute error between EEG and fNIRS recordings was reported as 0.58 and between EEG and multimodal recordings as 0.76. Between EEG and multimodal data types, one-way ANOVA testing yielded $p=3.2\times 10^{-3}$. Tukey posthoc comparisons indicated that EEG and multimodal data had significant differences, p<0.05. Multimodal recordings achieved sensitivity and specificity of 89.7% and 95.5%, respectively. The precision–recall curve confirmed this finding with multimodal EEG-fNIRS recordings having the highest values for both precision and recall.

### 3.3 Seizure Detection and Spatial Foci Localization

**Fig. 2:**
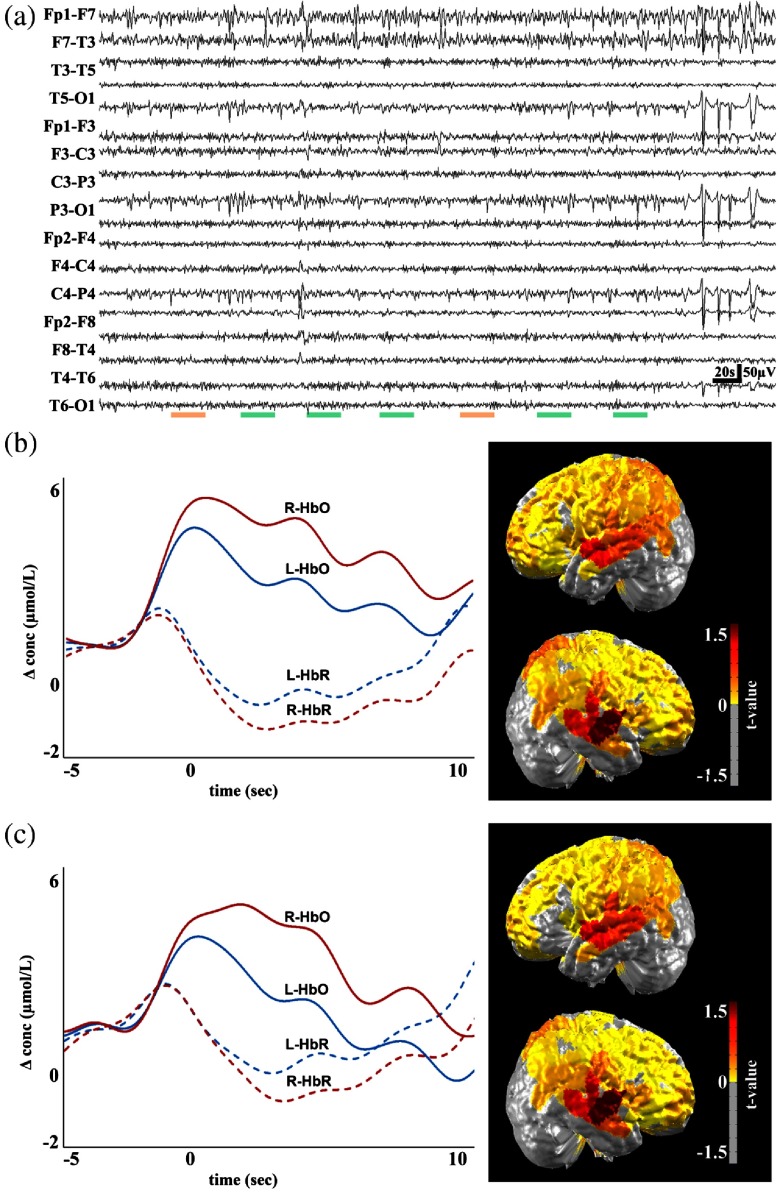
Multimodal recordings from patient 10, a 43-year-old male. On the day of the recording, the patient experienced multiple seizure events ranging from duration of 3 to 10 s, with an average duration of 7 s. The analyzed EEG recording is shown in (a), with the colored green bars representing seizure events and false positives denoted by orange horizontal lines. The hemodynamic response to marked events and network events (with false detections) and the corresponding cerebral topographic analysis are shown in (b) and (c). Red and blue curves represent oxygenated (HbO) and deoxygenated (HbR) hemoglobin, respectively. Solid red and blue and dashed red and blue lines correspond to the right (R- ) and left (L- ) side of the brain, respectively.

We further investigated our algorithm’s ability to classify signals correctly into seizure and nonseizure segments and localize classifications corresponding to the epileptogenic zone and relevant ictal processes. To this end, we performed analyses to determine if our algorithm’s outputs yielded similar cerebral localization results as traditional methods, primarily the general linear model (GLM).^9^ We first analyze the data on marked events and then performed GLM analysis on positive outputs from the network. In Fig. 2, green and orange bars denote true positive and false negative segments, respectively. The red and blue curves correspond to oxygenated and deoxygenated hemoglobin, respectively, and the hemodynamic curves from the right (solid lines) and left sides (dashed lines) of the epileptic foci are shown.

## 4 Discussion

The recent rapid technical advances in machine learning, particularly DL algorithms, have allowed for automated detection and prediction of anomalies in time series data. Similarly, these approaches hold promise for automated seizure detection with minimal processing of input data. In particular, input streams, such as EEG, fNIRS, and multimodal EEG-fNIRS, are well suited to be used in these algorithms. The LSTM model developed in this work was proven to be efficient in the task of seizure detection. Our architecture bypasses laborious hand feature selection and delivers good performance based on multimodal data input. LSTM-RNN architectures, like neural networks generally, can be a powerful tool, but they require long periods for training time, often require more data to train than other models, and can contain a large number of parameters to tune. The gradient vanishing problem and subsequent gradient exploration make training LSTMs difficult. Adaptive learning methods, particularly adaptive moment estimation (Adam), root mean square propagation (RMSProp), and adaptive gradient algorithms (AdaGrad), offer solutions to these gradient problems. Adam, which was used in this work, is an extension of and an optimization algorithm for stochastic gradient descent. Adam provides an optimization algorithm that can handle sparse gradients on noisy datasets, which is well suited for our purpose. Another technique used to handle noisy gradient estimates is to utilize mini-batches, as was done in our model. Smaller batches provide reduce computation time per update and offer faster model convergence.

Validation of our architecture on the standardized CHBMIT dataset yielded superior accuracy metrics (98.2%) compared to other studies using convolutional neural networks, support vector machines, and ANNs on the same dataset.^15^^,^^16^ Compared to other model architectures and related work in the literature, LSTMs offer a powerful framework for seizure detection. Particularly, the LSTM is well suited for time series data because it is well designed to extract patterns where the input data span over long sequences, a characteristic unique to seizure data. Once our model was validated on the CHBMIT standard dataset, we extended our model to multimodal data. Our implementation of a multilayered LSTM-RNN model for automated classification of multimodal EEG-fNIRS signals displays convergence and good performance metrics. Heuristic parameter tuning and the requirement of large datasets remain as limitations of our model. We in part solved this problem by collecting data points from multiple recordings allowing for a relatively large data corpus. This provides enough data variability to increase the power of the detection algorithm. Our experiments demonstrate that the hemodynamic profiles derived from fNIRS recordings provide discriminatory power in differentiating between seizure and nonseizure states. Furthermore, multimodal EEG-fNIRS data produced globally superior performance metrics when compared to stand-alone EEG or fNIRS data (Table 5). We obtained an improvement in performance when fNIRS recordings were stacked with the EEG data stream as compared to EEG data only. Our statistical analyses note significant differences in precision and recall metrics between EEG and multimodal data. The mean precision of EEG compared to multimodal data was 82.8 and 87.3, respectively. Likewise, the recall values between these data types were 85.2 and 89.7, respectively. Figure 2 suggests that our observations are consistent with our algorithm’s detections and those from analysis using the GLM. These findings suggest that deep neural models may eventually be a useful clinical tool in the demarcation of seizure zones for tailored medical therapy. Our analyses may also be useful in identifying the side of greater seizure susceptibility, and the localization derived from the network may potentially help guide epilepsy surgery and predict outcome postsurgery. Further, prospective studies with longer follow-up periods are needed to properly assess the utility of the model in this capacity. These changes might be indicative of preictal changes in the states of activity in localized neuronal networks and possibly beyond the ictal onset zone. Most patients suffering from epilepsy experience spikes, pre- and postseizure. This phenomenon was present in our dataset as well. Our algorithm has the potential to be extended to utilize interictal spikes as an additional feature, thereby, producing more comprehensive detection capabilities and providing a more complete clinical picture. This model can be particularly useful in situations in which a trained epileptologist is not readily available, in which paroxysmal cerebral electrical and hemodynamic changes may signal epileptic events. Our experimental findings have shown that combining fNIRS with EEG allows for improved seizure detection ability as compared to EEG alone, which could provide additional information on critically ill patients admitted in the ICU, thus improving detection of seizure (in addition to the other advantages of fNIRS in the ICU, such as monitoring of brain hypoxia).

**Table 5:** The overall classification result across all 10-folds for each data type. Multimodal data consistently provided superior results compared to stand-alone EEG or fNIRS data alone.

	Mean value post cross validation, k=10
Accuracy	
EEG	97.6±0.4 SD
fNIRS	97.0±0.7 SD
EEG-fNIRS	98.3±0.8 SD
Precision	
EEG	82.8±0.5 SD
fNIRS	80.7±0.6 SD
EEG-fNIRS	87.3±0.8 SD
Recall	
EEG	85.2±0.8 SD
fNIRS	81.3±0.6 SD
EEG-fNIRS	89.7±0.5 SD

Note: SD, standard deviation.

## 5 Conclusion

This study focused on determining the potential of fNIRS, a cost effective, portable neuroimaging technique in the detection of seizure events in multimodal EEG-fNIRS recordings. Our primary objective was to examine the enhanced capabilities that fNIRS signals provide for a seizure detection task, in particular when combined with EEG data in a multimodal framework, and our secondary objective was to utilize the power of neural networks for this task. For this study, we aimed to obtain strong and robust hemodynamic response signals. Toward this aim, we collected long-term continuous multimodal EEG-fNIRS data from 40 known epileptic patients comprising a total of 50 h of recordings. We proposed an LSTM-RNN model that is capable of learning explicit classes from human seizure data. Hyperparameter optimization and monitoring model validation loss (cross-entropy) to ensure network learning and reduce overfitting was a priority. Eventually, a multilayered RNN-LSTM neural network was designed to encode the sequential order of features using the rectified linear unit objective function. To examine the generative power of the LSTM-RNN model, we validated our model on a standard dataset followed by in-house data. Postvalidation, our recordings were scored and subsequent classes were formed from which a multilayered RNN-LSTM neural network was fed stand-alone EEG, stand-alone fNIRS data, and finally multimodal data. Our methodological approach proves its ability to automatically learn robust features from information contained in multimodal signals while conserving intrinsic waveform properties of seizure and nonseizure activity. Utilizing appropriate model hyperparameters, we performed model training, testing, and validation on a benchmarked scalp EEG dataset, which was followed by in-house EEG, fNIRS, and multimodal data from 40 epileptic patients. We explored the benefit that cerebral hemodynamic data provide for a seizure detection task in EEG-fNIRS neuroimaging data and we show that the addition of cerebral hemodynamics improves model performance when compared to EEG alone. Our model’s ability to learn the general representation of a seizure is showcased by cross-patient performance indicators as multimodal data reach performance metrics detailed in Table 5. Increased data collection, including different seizure types, can enhance our model’s performance and lend itself to increase generalizability. Furthermore, the neural network models proposed and characterized herein offer a promising framework for future investigations in early seizure detection. Since our proposed model correctly classifies sequences, this suggests automation of this process can enhance the diagnostic decision-making and treatment planning for epileptic patients. Our model has the potential to be extended to a real-time clinical monitoring system, in which trained clinical personnel are not readily accessible.
